# SH2 domains: modulators of nonreceptor tyrosine kinase activity

**DOI:** 10.1016/j.sbi.2009.10.001

**Published:** 2009-12

**Authors:** Panagis Filippakopoulos, Susanne Müller, Stefan Knapp

**Affiliations:** 1 Structural Genomics Consortium, University of Oxford, Old Road Campus Research Building, Roosevelt Drive, Headington, Oxford OX3 7DQ, UK; 2 Department of Clinical Pharmacology, University of Oxford, Old Road Campus Research Building, Roosevelt Drive, Headington, Oxford OX3 7DQ, UK

## Abstract

The Src homology 2 (SH2) domain is a sequence-specific phosphotyrosine-binding module present in many signaling molecules. In cytoplasmic tyrosine kinases, the SH2 domain is located N-terminally to the catalytic kinase domain (SH1) where it mediates cellular localization, substrate recruitment, and regulation of kinase activity. Initially, structural studies established a role of the SH2 domain stabilizing the inactive state of Src family members. However, biochemical characterization showed that the presence of the SH2 domain is frequently required for catalytic activity, suggesting a crucial function stabilizing the active state of many nonreceptor tyrosine kinases. Recently, the structure of the SH2–kinase domain of Fes revealed that the SH2 domain stabilizes the active kinase conformation by direct interactions with the regulatory helix αC. Stabilizing interactions between the SH2 and the kinase domains have also been observed in the structures of active Csk and Abl. Interestingly, mutations in the SH2 domain found in human disease can be explained by SH2 domain destabilization or incorrect positioning of the SH2. Here we summarize our understanding of mechanisms that lead to tyrosine kinase activation by direct interactions mediated by the SH2 domain and discuss how mutations in the SH2 domain trigger kinase inactivation.

## Introduction

Protein kinase activity is tightly controlled in eukaryotic cells. To guarantee quick and specific propagation of cellular signals most kinases are locked in an inactive state that can be rapidly activated by interaction with regulatory elements such as interacting proteins or domains located outside the catalytic domain as well as post-translational modifications. In addition, selectivity of signaling is dramatically enhanced by localizing kinases to signaling complexes and through selective targeting of substrates via interactions with secondary substrate recruitment sites.

SH2 domains represent the largest class of pTyr-selective recognition domains in the human proteome [1, 2]. In cytoplasmic tyrosine kinases the arrangement of an N-terminal SH2 domain followed by a kinase domain is highly conserved in all family members and probably evolved as an invariant signaling unit early in evolution with the occurrence of tyrosine phosphorylation. The conserved SH2–kinase domain unit has already been reported to be present in the unicellular choanoflagellate *Monosiga brevicollis*, a primitive common ancestor of multicellular organisms [3^•^]. It is believed that this domain arrangement initially served to target kinases to their substrates [4]. With the increasing complexity of signaling in multicellular organisms the flanking SH2 domain assumed other regulatory functions such as allosteric regulation of the kinase catalytic domain [5]. Interestingly, already in *M. brevicollis* the Csk ortholog (MbCSK) phosphorylates the Src ortholog MBSrc but the autoinhibitory role of this phosphorylation event, which is a hallmark of Src kinases inactivation, had not yet developed in *Monosiga* and evolved most likely much later within the metazoan lineage [3^•^].

The selectivity of SH2 domains for their pTyr substrates has been investigated using directed phosphopeptide library screening and a large number of biophysical and biochemical studies [6, 7, 8, 9, 10]. These studies revealed that most of the binding affinity (∼50%) is attributed to the phosphate moiety of the pTyr residue while residues in positions from −2 to +4, relative to the phosphotyrosine, modulate binding specificity. However, structural studies revealed larger contact interfaces spanning from residues −6 to +6 in some cocrystal structures [11, 2].

**Figure 1:**
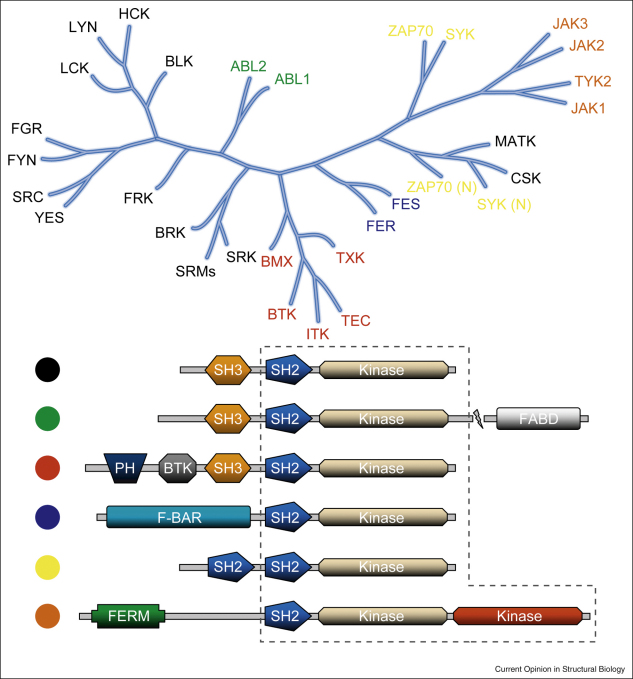
Phylogenetic tree based on SH2 domain sequence and domain organization in nonreceptor tyrosine kinases. The different types of domain architectures shown in the lower panel are highlighted by different colors and in the phylogenetic tree the name of kinases with a certain domain organization are colored accordingly. The SH2–kinase unit is indicated by a dashed line. In Jak kinases this unit involves probably both kinase domains, which seem to form a compact structural unit [34].

In nonreceptor tyrosine kinases the conserved SH2–kinase unit is flanked by a number of additional domains which may comprise the N-terminal flanking region, a Src homology 3 (SH3) domain, a second SH2 domain, a sequence of PH–BTK–SH3 domains, a ‘Four-point-one, Ezrin, Radixin, Moesin’ (FERM) domain, or an F-BAR domain. In Abl kinases the C-terminus is extended containing an F-actin binding domain (FABD) (Figure 1).

## SH2 domain interactions leading to kinase inactivation

The mechanism of autoinhibition of Src family members has been elucidated by a number of high-resolution X-ray crystallography studies [12, 13••, 14]. The key event of Src inactivation is that the C-terminal Tyr527 in Src, or a corresponding tyrosine in other family members, is phosphorylated by Csk [15]. The phosphorylated Tyr527 binds intramolecularly to the SH2 domain resulting in the formation of a compact inactive state. This conformation is additionally stabilized by interactions of the SH3 domain with proline rich sequences connecting the SH2 domain and the kinase domain. Dephosphorylation of Tyr527 converts Src to an open active conformation and triggers autophosphorylation of the activation segment residue Tyr416 to yield a fully activated enzyme. Molecular dynamics simulation and mutational analysis showed that the SH2 and SH3 domains of the inactive kinases are tightly coupled by the connecting linker stabilizing the inactive state in Src [16]. Interestingly also the isolated SH3–SH2 domains of Fyn maintain the relative orientation observed in the closed inactive kinase conformation of Src [17]. In contrast the orientations of the SH2–SH3 domain differed significantly in Lck which has a diverse linker sequence suggesting that the linker between the SH2 and SH3 domains affects the potential of the regulatory fragments to repress kinase activity.

The crystal structure of the open active kinase conformation of Src confirmed that the SH2/SH3 domain is rotated away from the kinase domain but it retains its ‘clamp’ structure in which the SH3 domain remains bound to the SH2 linker [18^•^]. The open conformation of the active Src kinase domain observed by crystallography has been supported in solution by small-angle scattering [19].

Many aspects of Src inactivation are conserved in Abl kinases, which are also autoinhibited by intramolecular interactions of the SH2/SH3 domains. However, autoinhibition in Abl is phosphotyrosine independent [20, 21]. The lack of a residue corresponding to Src pTyr527 in Abl is compensated for by an N-terminal myristoyl modification, which tightly binds to the base of the kinase domain lower lobe. This binding event induces bending of the C-terminal helix of the kinase domain and binding of the SH2 to the kinase domain. Differences between the allosteric regulation of Abl and Src result in distinct activation and deactivation kinetics and pronounced selectivity of the kinase inhibitor Gleevec for the Abl inactive state [22].

Zap70 and the closely related kinase Syk contain two N-terminal SH2 domains instead of the Src SH3–SH2 domain arrangement (Figure 1). In the inactive state two helices of the inter SH2 linker dock onto the C-terminal lobe helices I and E [23^••^]. The SH2–SH2 domain linker region contains two tyrosine residues, which form aromatic stacking interactions with the kinase domain. Phosphorylation of these two residues activates Zap70 and allows binding of immunoreceptor tyrosine-based activation motifs (ITAM), which are not recognized by the inactive complex. A similar mechanism has been suggested for Syk [24]. Interestingly, the SH2 domains in Zap70 have accessible pTyr binding sites but high affinity binding to di-phosphorylated ITAM is disrupted due to incompatible domain orientation that disrupts the phosphotyrosine-binding pocket of the N-terminal SH2 domain.

## SH2 domain interactions stabilizing the kinase active state

In the structure of active Src the SH2 domain does not interact with the kinase domain and has no influence on the active state of this kinase. Indeed, deletion of the entire N-terminal region comprising both the SH3 and SH2 domains has little effect on Src kinase activity [25].

The situation is very different in Csk where the deletion of the SH3 and SH2 domains results in a drastic reduction of enzymatic activity [26, 27, 28]. Detailed mutagenesis experiments showed that several residues in the SH2–kinase linker region are important for catalytic activity [27]. The crystal structure of full-length Csk revealed a well-defined interaction site of the SH2 and SH3 domains on either side of the N lobe and biochemical studies demonstrate that these interactions stimulate the catalytic activity of Csk [29^•^].

SAXS analysis of active Brutons tyrosine kinase (Btk), also revealed close proximity of the SH2 domain to the N lobe of the kinase domain [30^•^]. However, in contrast to Csk the SH3–SH2–kinase domains assume an extended conformation suggesting a well-defined and stable arrangement of the domains distinct from the flexible tethering observed in active c-Src. The presence of the SH2 domain as well as the linker region between SH2 and kinase domain has been shown to be necessary for activity of Itk and, by analogy, also for the activity of other Tec family members (Tec, Btk, Txk and Bmx). Site-directed mutagenesis of residues in the conserved linker region of Itk and Btk suggested a strong positive effect on kinase activity [31, 32]. Little is known about the function of the SH2 domain in Jak kinases. Some members of that family are lacking the invariant arginine residue that plays an important role in coordinating phosphotyrosine ligands [33]. It has been shown that Jak function is independent of phosphotyrosine binding and it is assumed that the Jak SH2 domain plays a structural role rather than serving as a substrate recruitment module and its influence on Jak kinase activity is unknown [34].

Also the active state of Abl is stabilized by a tight contact of the SH2 domain with the upper kinase lobe [35]. Mutation of residues located in the interface significantly impaired Abl catalytic activity and the SH2–kinase unit of Abl is about fourfold more active than the isolated kinase domain [35, 36••]. The interaction of the SH2 domain with the upper lobe of the kinase domain has been supported by deuterium exchange studies using mass spectroscopy [37]. The precise mechanism by which the SH2 domain activates kinase function by this interaction remains enigmatic.

**Figure 2:**
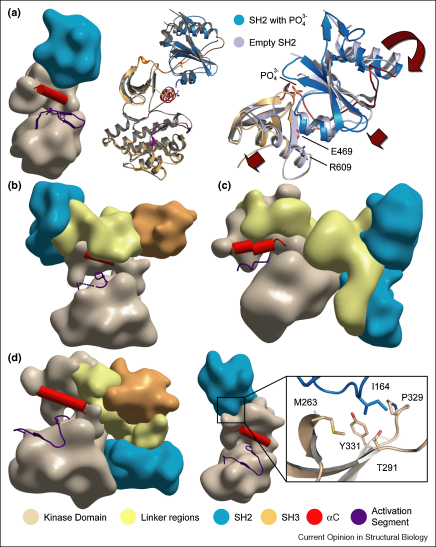
Interfaces between kinase domains and SH2/linker. **(a)** SH2–kinase domain arrangement in Fes. Shown is the orientation of the domains in low-resolution surface representation in the left panel. Structural details upon inactivation are shown in the middle panel and a detailed view of the SH2–αC interaction is shown in the right panel. Key residues of the interaction are highlighted. **(b)** Domain organization in active Csk. **(c)** Domain organization in inactive Zap70. **(d)** Domain packing in inactive Abl (left panel), active Abl (middle panel), and detailed view of the interaction between the SH2 domain and the kinase domain (right panel). As indicated in the lower panel in all surface representations SH3 domains are shown in orange, SH2 domains in blue, linker regions between SH2 and kinase domain in yellow, helix αC in red, activation segments in purple and the kinase domains are shown in beige.

In Fes/Fps insertion mutagenesis identified the SH2 domain as a strongly positive regulator of kinase activity. Dipeptide insertions in the N-terminal region of the v-Fps SH2 domain cause a severe loss of both kinase and transforming functions, suggesting that the conformation of the SH2 domain is important for the catalytic properties of the adjacent kinase domain [38••, 39]. The stable character of the SH2–kinase unit was further demonstrated by limited trypsin digestion, which yielded a protease-resistant 45 kDa fragment containing both the SH2 and kinase domains [40]. The crystal structure of active Fes revealed a compact structural unit of the SH2–kinase domain in which the catalytically important helix C is positioned in tight contact with the SH2 domain [36^••^]. In contrast to Src family members the SH2 domain moves away from the kinase domain in inactive Fes. The separation of the SH2 and kinase domains and absence of a ligand occupying the SH2 binding site result in partial unfolding of the SH2 domain and higher mobility of αC in the kinase domain. Thus, binding of a primed substrate to the Fes SH2 domain is an early step in Fes activation that results in allosteric regulation of the catalytic domain. The different scenarios of kinase activation and inactivation by SH2 domains are summarized in Figure 2.

## Role of destabilizing mutations in the SH2 domain and linker region in disease

The SH2 domain is a key regulator of allosteric kinase regulation and may play a role in both the inactive as well as the active states of tyrosine kinases. It is therefore no surprise that mutations have been identified in patients that compromise SH2 function and as a consequence kinase activity. A recent survey found that the majority of the disease-causing missense mutations in SH2 domains affect functionally important amino acids (71%) either directly involved in ligand binding, or residues that mediate interactions with the catalytic domain. The remaining missense mutations are located distantly from the SH2 domain and presumably affect its stability [41]. In Fes, the structure of the SH2–kinase unit revealed a mechanism that explains how destabilization of the SH2 domain can affect enzymatic activity by relocating the SH2 domain and effectively losing contact with the kinase domain. In the closely related kinase Fer a mutation of the highly conserved residue (W460C) located in the N-terminus of the SH2 domain has been detected in lung cancer [42]. Trp460 is part of the hydrophobic core of the SH2 domain and is not involved in pTyr binding. This residue is also conserved in Fes and its location suggests that it is an essential residue for SH2 domain stability resulting in kinase inactivation and most likely rapid degradation of the protein in the cellular environment. Similarly, the missense mutation P80Q in Zap70 causes severe combined immunodeficiency (SCID) by reducing the stability of the Zap70 SH2 domain associated with rapid degradation of the protein [43]. A destabilizing mutation (E481G) causing SCID has also been reported for the Jak3 SH2 domain [44].

**Figure 3:**
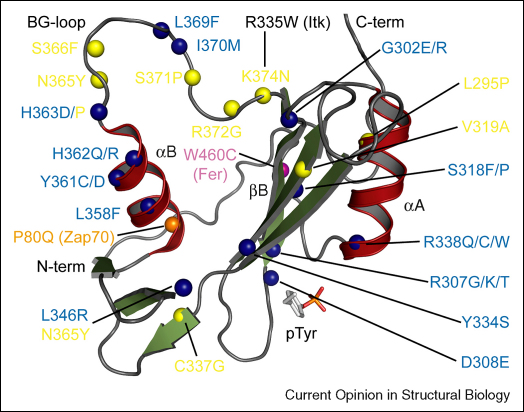
Location of mutations identified in human disease projected onto the structure of the Btk SH2 domain. Data used in the figure were taken from Ref. [45^•^]. Mutations that destabilize the SH2 domain are shown in yellow and those affecting pTyr binding are highlighted in blue. The locations of the destabilizing mutation R335W in Itk, P80Q in Zap70, and W460C in Fer are also shown. The major structural elements harboring mutations as well as the C-termini and N-termini are labeled.

One of the best studied examples of disease-causing mutations in SH2 domains is the Tec family member Btk. Mutations in the SH2 domain of this kinase cause X-linked agammaglobulinemia (XLA), a prototypical humoral immunodeficiency characterized by low levels of circulating B cells and a drastic reduction in serum concentrations of immunoglobulins [45^•^]. So far more than 30 missense mutations in the BTK SH2 domain have been described in XLA patients and the effects of these mutations have been analyzed by a large number of *in vitro* and *in vivo* studies [45•, 46, 47, 48]. About 20 mutations are directly involved in ligand binding and disturb the interaction with signaling partners, like SLAM family members and SH2D1A. The remaining mutations are located outside the ligand-binding pocket and affect protein stability, resulting in reduced half-life of the mutated protein in cells. Recently a mutation in the SH2 domain of the Tec family member Itk has been linked to severe immune dysfunction and therapy-resistant Epstein–Barr virus (EBV)-positive B cell proliferation. The mutation R335W is located in the BG loop of the Itk SH2 domain. Arg335 is not involved in pTyr binding and mutation to tryptophan most likely causes instability of the SH2 domain and significantly reduced protein levels in patients as a result of increased protein degradation [49^•^]. Since a functional SH2 domain is necessary for enzymatic activity it is likely that also kinase activity is compromised in mutants that destabilize the SH2 domain in Tec family members [31, 32]. A summary of mutants identified in human disease and their location is shown in Figure 3.

Structural data and mutations found in human disease that lead to SH2 domain destabilization suggest a pivotal role of the SH2 domain in structural stability and in the stabilization of the active kinase conformation for a large number of nonreceptor tyrosine kinases. We hope that further structural and functional studies on multidomain constructs of tyrosine kinases will shed further light on the diverse mechanisms of kinase activation for this important group of enzymes.

## References and recommended reading

Papers of particular interest, published within the period of review, have been highlighted as:

• of special interest
•• of outstanding interest

## References

[1] Pawson T., Gish G.D., Nash P. (2001). SH2 domains, interaction modules and cellular wiring. Trends Cell Biol.

[2] Liu B.A., Jablonowski K., Raina M., Arce M., Pawson T., Nash P.D. (2006). The human and mouse complement of SH2 domain proteins-establishing the boundaries of phosphotyrosine signaling. Mol Cell.

[3•] Li W., Young S.L., King N., Miller W.T. (2008). Signaling properties of a non-metazoan Src kinase and the evolutionary history of Src negative regulation. J Biol Chem.

[4] Mayer B.J., Hirai H., Sakai R. (1995). Evidence that SH2 domains promote processive phosphorylation by protein-tyrosine kinases. Curr Biol.

[5] Kuriyan J., Eisenberg D. (2007). The origin of protein interactions and allostery in colocalization. Nature.

[6] Grucza R.A., Bradshaw J.M., Futterer K., Waksman G. (1999). SH2 domains: from structure to energetics, a dual approach to the study of structure–function relationships. Med Res Rev.

[7] Bradshaw J.M., Waksman G. (1999). Calorimetric examination of high-affinity Src SH2 domain-tyrosyl phosphopeptide binding: dissection of the phosphopeptide sequence specificity and coupling energetics. Biochemistry.

[8] Bradshaw J.M., Mitaxov V., Waksman G. (1999). Investigation of phosphotyrosine recognition by the SH2 domain of the Src kinase. J Mol Biol.

[9] Songyang Z., Shoelson S.E., Chaudhuri M., Gish G., Pawson T., Haser W.G., King F., Roberts T., Ratnofsky S., Lechleider R.J. (1993). SH2 domains recognize specific phosphopeptide sequences. Cell.

[10] Machida K., Thompson C.M., Dierck K., Jablonowski K., Karkkainen S., Liu B., Zhang H., Nash P.D., Newman D.K., Nollau P. (2007). High-throughput phosphotyrosine profiling using SH2 domains. Mol Cell.

[11] Hu J., Hubbard S.R. (2005). Structural characterization of a novel Cbl phosphotyrosine recognition motif in the APS family of adapter proteins. J Biol Chem.

[12] Xu W., Harrison S.C., Eck M.J. (1997). Three-dimensional structure of the tyrosine kinase c-Src. Nature.

[13••] Xu W., Doshi A., Lei M., Eck M.J., Harrison S.C. (1999). Crystal structures of c-Src reveal features of its autoinhibitory mechanism. Mol Cell.

[14] Sicheri F., Moarefi I., Kuriyan J. (1997). Crystal structure of the Src family tyrosine kinase Hck. Nature.

[15] Okada M., Nakagawa H. (1989). A protein tyrosine kinase involved in regulation of pp60c-src function. J Biol Chem.

[16] Young M.A., Gonfloni S., Superti-Furga G., Roux B., Kuriyan J. (2001). Dynamic coupling between the SH2 and SH3 domains of c-Src and Hck underlies their inactivation by C-terminal tyrosine phosphorylation. Cell.

[17] Arold S.T., Ulmer T.S., Mulhern T.D., Werner J.M., Ladbury J.E., Campbell I.D., Noble M.E. (2001). The role of the Src homology 3-Src homology 2 interface in the regulation of Src kinases. J Biol Chem.

[18•] Cowan-Jacob S.W., Fendrich G., Manley P.W., Jahnke W., Fabbro D., Liebetanz J., Meyer T. (2005). The crystal structure of a c-Src complex in an active conformation suggests possible steps in c-Src activation. Structure.

[19] Bernado P., Perez Y., Svergun D.I., Pons M. (2008). Structural characterization of the active and inactive states of Src kinase in solution by small-angle X-ray scattering. J Mol Biol.

[20] Nagar B., Hantschel O., Young M.A., Scheffzek K., Veach D., Bornmann W., Clarkson B., Superti-Furga G., Kuriyan J. (2003). Structural basis for the autoinhibition of c-Abl tyrosine kinase. Cell.

[21] Hantschel O., Nagar B., Guettler S., Kretzschmar J., Dorey K., Kuriyan J., Superti-Furga G. (2003). A myristoyl/phosphotyrosine switch regulates c-Abl. Cell.

[22] Schindler T., Bornmann W., Pellicena P., Miller W.T., Clarkson B., Kuriyan J. (2000). Structural mechanism for STI-571 inhibition of Abelson tyrosine kinase. Science.

[23••] Deindl S., Kadlecek T.A., Brdicka T., Cao X., Weiss A., Kuriyan J. (2007). Structural basis for the inhibition of tyrosine kinase activity of ZAP-70. Cell.

[24] Zhang Y., Oh H., Burton R.A., Burgner J.W., Geahlen R.L., Post C.B. (2008). Tyr130 phosphorylation triggers Syk release from antigen receptor by long-distance conformational uncoupling. Proc Natl Acad Sci U S A.

[25] Xu B., Miller W.T. (1996). Src homology domains of v-Src stabilize an active conformation of the tyrosine kinase catalytic domain. Mol Cell Biochem.

[26] Sondhi D., Cole P.A. (1999). Domain interactions in protein tyrosine kinase Csk. Biochemistry.

[27] Shekhtman A., Ghose R., Wang D., Cole P.A., Cowburn D. (2001). Novel mechanism of regulation of the non-receptor protein tyrosine kinase Csk: insights from NMR mapping studies and site-directed mutagenesis. J Mol Biol.

[28] Cole P.A., Shen K., Qiao Y., Wang D. (2003). Protein tyrosine kinases Src and Csk: a tail's tale. Curr Opin Chem Biol.

[29•] Ogawa A., Takayama Y., Sakai H., Chong K.T., Takeuchi S., Nakagawa A., Nada S., Okada M., Tsukihara T. (2002). Structure of the carboxyl-terminal Src kinase, Csk. J Biol Chem.

[30•] Marquez J.A., Smith C.I., Petoukhov M.V., Lo Surdo P., Mattsson P.T., Knekt M., Westlund A., Scheffzek K., Saraste M., Svergun D.I. (2003). Conformation of full-length Bruton tyrosine kinase (Btk) from synchrotron X-ray solution scattering. EMBO J.

[31] Joseph R.E., Min L., Andreotti A.H. (2007). The linker between SH2 and kinase domains positively regulates catalysis of the Tec family kinases. Biochemistry.

[32] Guo S., Wahl M.I., Witte O.N. (2006). Mutational analysis of the SH2–kinase linker region of Bruton's tyrosine kinase defines alternative modes of regulation for cytoplasmic tyrosine kinase families. Int Immunol.

[33] Radtke S., Haan S., Jorissen A., Hermanns H.M., Diefenbach S., Smyczek T., Schmitz-Vandeleur H., Heinrich P.C., Behrmann I., Haan C. (2005). The Jak1 SH2 domain does not fulfill a classical SH2 function in Jak/STAT signaling but plays a structural role for receptor interaction and up-regulation of receptor surface expression. J Biol Chem.

[34] Haan C., Kreis S., Margue C., Behrmann I. (2006). Jaks and cytokine receptors — an intimate relationship. Biochem Pharmacol.

[35] Nagar B., Hantschel O., Seeliger M., Davies J.M., Weis W.I., Superti-Furga G., Kuriyan J. (2006). Organization of the SH3–SH2 unit in active and inactive forms of the c-Abl tyrosine kinase. Mol Cell.

[36••] Filippakopoulos P., Kofler M., Hantschel O., Gish G.D., Grebien F., Salah E., Neudecker P., Kay L.E., Turk B.E., Superti-Furga G. (2008). Structural coupling of SH2–kinase domains links Fes and Abl substrate recognition and kinase activation. Cell.

[37] Iacob R.E., Pene-Dumitrescu T., Zhang J., Gray N.S., Smithgall T.E., Engen J.R. (2009). Conformational disturbance in Abl kinase upon mutation and deregulation. Proc Natl Acad Sci U S A.

[38••] Stone J.C., Atkinson T., Smith M., Pawson T. (1984). Identification of functional regions in the transforming protein of Fujinami sarcoma virus by in-phase insertion mutagenesis. Cell.

[39] Sadowski I., Stone J.C., Pawson T. (1986). A noncatalytic domain conserved among cytoplasmic protein-tyrosine kinases modifies the kinase function and transforming activity of Fujinami sarcoma virus P130gag-fps. Mol Cell Biol.

[40] Weinmaster G., Zoller M.J., Smith M., Hinze E., Pawson T. (1984). Mutagenesis of Fujinami sarcoma virus: evidence that tyrosine phosphorylation of P130gag-fps modulates its biological activity. Cell.

[41] Vihinen M., Nilsson L., Smith C.I. (1994). Structural basis of SH2 domain mutations in X-linked agammaglobulinemia. Biochem Biophys Res Commun.

[42] Richardson C.J., Gao Q., Mitsopoulous C., Zvelebil M., Pearl L.H., Pearl F.M. (2009). MoKCa database — mutations of kinases in cancer. Nucleic Acids Res.

[43] Matsuda S., Suzuki-Fujimoto T., Minowa A., Ueno H., Katamura K., Koyasu S. (1999). Temperature-sensitive ZAP70 mutants degrading through a proteasome-independent pathway. Restoration of a kinase domain mutant by Cdc37. J Biol Chem.

[44] Candotti F., Oakes S.A., Johnston J.A., Giliani S., Schumacher R.F., Mella P., Fiorini M., Ugazio A.G., Badolato R., Notarangelo L.D. (1997). Structural and functional basis for JAK3-deficient severe combined immunodeficiency. Blood.

[45•] Lappalainen I., Thusberg J., Shen B., Vihinen M. (2008). Genome wide analysis of pathogenic SH2 domain mutations. Proteins.

[46] Fiorini M., Franceschini R., Soresina A., Schumacher R.F., Ugazio A.G., Rossi P., Plebani A., Notarangelo L.D. (2004). BTK: 22 novel and 25 recurrent mutations in European patients with X-linked agammaglobulinemia. Hum Mutat.

[47] Tzeng S.R., Pai M.T., Lung F.D., Wu C.W., Roller P.P., Lei B., Wei C.J., Tu S.C., Chen S.H., Soong W.J. (2000). Stability and peptide binding specificity of Btk SH2 domain: molecular basis for X-linked agammaglobulinemia. Protein Sci.

[48] Hashimoto S., Tsukada S., Matsushita M., Miyawaki T., Niida Y., Yachie A., Kobayashi S., Iwata T., Hayakawa H., Matsuoka H. (1996). Identification of Bruton's tyrosine kinase (Btk) gene mutations and characterization of the derived proteins in 35 X-linked agammaglobulinemia families: a nationwide study of Btk deficiency in Japan. Blood.

[49•] Huck K., Feyen O., Niehues T., Ruschendorf F., Hubner N., Laws H.J., Telieps T., Knapp S., Wacker H.H., Meindl A. (2009). Girls homozygous for an IL-2-inducible T cell kinase mutation that leads to protein deficiency develop fatal EBV-associated lymphoproliferation. J Clin Invest.

